# Validation of the Perception of eHealth Technology Scale in Chinese Brief (PETS-C Brief) in Nurses: Survey Study

**DOI:** 10.2196/79594

**Published:** 2025-11-05

**Authors:** Ayisha Jilili, Xue Weng, Palida Maimaiti, Liwen Liao, Sheng Zhi Zhao, Lin Wang, Ningyuan Guo

**Affiliations:** 1 School of Nursing, Shanghai Jiao Tong University, 227 South Chongqing Road, Shanghai, 200025, China, 86 63846590; 2 School of Nursing, Xinjiang Medical University, Urumqi, China; 3 Institute of Advanced Studies in Humanities and Social Sciences, Beijing Normal University, Zhuhai, China; 4 Department of Nursing, Shanghai Mental Health Center, Shanghai, China; 5 School of Nursing, University of Hong Kong, Hong Kong, China (Hong Kong)

**Keywords:** eHealth, perception of eHealth technology scale, validation, nurses, eHealth technology scale

## Abstract

**Background:**

eHealth technologies have shown promise in improving the accessibility and quality of nursing research and practice. Little is known about nurses’ perception of eHealth technology that are prerequisites for the implementation of eHealth-based nursing care.

**Objective:**

We aimed to confirm the factor structure and examine the validity and reliability of the novel 19-item Perception of eHealth Technology Scale in Chinese Brief (PETS-C Brief) in Chinese nurses. The associations of sociodemographic and working-related characteristics with PETS-C Brief scores were investigated.

**Methods:**

Participants were 1409 nurses (96.8% female; mean age 34.6, SD 8.6 y) working in hospital or community settings in Shanghai, China. Confirmatory factor analysis was conducted to verify the previously reported four-factor structure of PETS-C Brief. Cronbach α was calculated for internal consistency reliability. One-month test–retest reliability was assessed in 123 participants completing the one-month follow-up survey. Associations of sociodemographic and working-related characteristics (ie, years of employment, professional title, and setting) with PETS-C Brief scores were analyzed using multivariable linear regression. Known-group validity was assessed by examining the associations of age and educational attainment with PETS-C Brief scores.

**Results:**

The goodness-of-fit of the four-factor PETS-C Brief was shown to be acceptable (comparative fit index [CFI]=0.95, standardized root mean squared residual [SRMR]=0.065, root mean square error of approximation [RMSEA]=0.074). The scale showed a good internal consistency reliability (Cronbach *α*=0.91) and one-month test–retest reliability (intraclass correlation coefficient=0.68, 95% CI: 0.55, 0.78). Known-group validity was supported by the inverse association of age with PETS-C Brief scores (*P*=.002) and positive association of educational attainment with PETS-C Brief scores (*P* for trend=.043). No significant associations were observed between working-related characteristics and PETS-C Brief scores.

**Conclusions:**

Our validation study supported the four-factor structure of PETS-C Brief with satisfactory validity and reliability in Chinese nurses, suggesting the scale could be deployed for assessing perception of eHealth technology. Future studies with larger sample, random sampling, and in other cultural settings are warranted to increase the generalizability.

## Introduction

eHealth technologies refers to the use of information and communication technologies (eg, internet, websites, smartphones, monitoring devices, applications) in health-related fields, including health surveillance, services, education, and research [1]. eHealth technologies have shown promise in improving the accessibility and quality of health care, such as in chronic disease management [2] and health behavior promotion [3]. Nurses, as the largest component of the health care workforce, have been the majority of eHealth technology users [4-6]. Benefits of nurses’ use of eHealth technologies were evident on improving patients’ health outcomes and quality of life in randomized controlled trials, systematic reviews, and meta-analyses [7-9]. Nurses’ use of eHealth technologies was also associated with higher job satisfaction [10]. Nurses’ perception of eHealth technologies can be a crucial factor for promoting use, which may influence their acceptance of eHealth technologies [11], quality of care [12], and patients’ engagement [13].

Perception of eHealth technologies may involve various aspects, such as digital literacy [12], knowledge, acceptance, attitude, and the potential promise and challenges of its use [14]. With the popularity of eHealth devices and applications in medical settings, knowledge and acceptance has been increasingly measured [15]. However, few studies have measured a broader perception of advantages, challenges, and even risks. The eHealth Literacy Scale (eHEALS), one of the earliest developed and most widely used measurements, was designed to assess the perceived skills to engage in eHealth [16]. However, eHEALS focuses primarily on consumers, such as patients receiving eHealth-based care. The Technology Acceptance Model (TAM) was initially developed to predict the use or acceptance of information technology in work [17] and then was adapted in the health care context specifically [18]. However, TAM may benefit from several additions and modifications during the adaption process. For example, personal productivity was a measure of usefulness, one of the constructs in TAM, may not be meaningful and sufficient in the health care context [18]. Many other studies have used self-made questionnaires to measure the perception of eHealth technologies. For example, a questionnaire was designed to assess UK students’ perception of electronic health records, including knowledge and proficiency in digital clinical systems, pretraining personal use and trained professional use of social media, and degree of usefulness for training [19]. But the validity and reliability of such self-made questionnaires were unclear.

The Perception of eHealth Technology Scale in Chinese Brief (PETS-C Brief) [14] was adapted from a 40-item instrument initially developed for assessing knowledge and perceptions of telemedicine technology in clinicians in Iran [20]. By adapting the instrument in Chinese context, Kwan and colleges modified and translated the items into Chinese. A broader concept of “eHealth technology” was used to replace “telemedicine” during modification and translation. Exploratory factor analysis (EFA) and item analysis were then conducted to reduce the number of items and identifying factors. A four-factor structure with 19 items was yielded, which was supported by satisfactory indices in confirmatory factor analysis (CFA). The four factors refer to knowledge of eHealth technology, perception of the advantages of eHealth technology, perception of the disadvantages of eHealth technology, and perception of eHealth applications [14]. However, validity and reliability of the PETS-C Brief in Chinese nurses was understudied, though results of which can complement those of factor analysis for supporting the scale quality.

In 2019, the Chinese government has issued a notice promoting the pilot program of the Internet Plus Nursing Service, defined as a health care service using internet and various eHealth technologies provided by registered nurses in hospital or community settings [21]. In 2020, for the purpose of this program’s nationwide spread, a Notice on Furthering the Pilot Project of Internet Plus Nursing Service was officially established [22]. However, one of the major challenges for implementing Internet Plus Nursing Service was limited participation by nurses [23]. Understanding nurses’ perception of eHealth technologies can be the first step for increasing nurses’ participation. But a standardized measurement is lacking. One study using PETS-C Brief has identified educational attainment as a determinant of perception of eHealth technology in Chinese nurses and nursing students [11]. Other studies on the digital divide in nurses suggested that educational attainment [24] and age [25,26] could influence nurses’ perception of eHealth technologies. Identifying factors affecting perception of eHealth technology is needed for developing targeted interventions and training programs promoting the Internet Plus Nursing Service in China. Hence, our study had three aims: first, to confirm the factor construct of PETS-C Brief reported in the previous study [11]; second, to examine the validity and reliability of PETS-C Brief; last, to investigate the associations of sociodemographic and working-related characteristics with PETS-C Brief scores.

## Methods

### Design, Setting and Sample

The study was conducted among nurses working in hospital or community settings using convenience sampling with open recruitment in Shanghai, China. At baseline, advertisements for the study were distributed through WeChat, one of the most popular social media in China. Interested nurses entered the study by scanning a QR code, and peer sharing and referral was used to increase the response rate. Eligibility was assessed using an inclusion and exclusion form on the first page of the survey. Inclusion criteria were age ≥18 years; registered nurses; aware of the content of the survey and willing to participate. Registered nurses are individuals who have completed relevant nursing professional education, obtained a nursing qualification certificate, and are registered and legally licensed to practice in medical institutions including hospitals and community settings in China. Those with any clinical diagnosis of mental health disorders were excluded. After the eligibility page, participants who clicked “I have read the informed consent form carefully and agree to participate” could respond to survey items. Implied consent to participate was indicated when participants provided responses to survey items. For assessing the one-month test-retest reliability, short massages containing a follow-up survey link were sent to participants, using telephone numbers collected at baseline; their names were not asked. The survey at baseline and one-month follow-up were programmed to allow only one completion per device to prevent duplicate submissions.

Sample size was calculated based on the requirement for conducting CFA. A total sample of 1000 participants is regarded as “excellent” in the statistical viewpoints and previously published literature [27]. For the sample of follow-up survey, a minimal sample size of 100 was recommended for a high reliability [28].

### Measurements

PETS-C brief was adapted from a 40-item instrument initially developed for assessing knowledge and perceptions of Telemedicine Technology in clinicians in Iran, each item was on a five-point Likert scale [20]. The original instrument included six factors as follows: knowledge (7 items), perception of the advantages (7 items), perception of the disadvantages (8 items), perception of necessity (6 items), perception of ease of usage (6 items), and perception of security (6 items). Kwan and colleges adapted the instrument in Chinese nurses through item modification and translation, and a broader concept of eHealth technology was used [14]. A four-factor structure with 19 items was yielded by conducting EFA and CFA. The four factors were knowledge (4 items), perception of the advantages (4 items), perception of the disadvantages (6 items), and perception of eHealth applications (5 items) [14]. Each item of PETS-C brief was scored on a five-point Likert scale from 1=“very low” to 5=“very high.” Total scores range 19‐95, with higher scores indicating better perceptions of eHealth technology. PETS-C Brief was set as a compulsory question, thereby no data were missing.

Sociodemographic characteristics included sex, age, educational attainment, marital status, monthly household income. Younger age [25,26] and higher educational attainment [24] were suggested as potential factors influencing nurses’ perception of eHealth technologies. Apart from the two variables, the selection of sex, marital status, and monthly household income was consistent with studies on Chinese nurses’ working experiences with Internet Plus Nursing Service for result comparison [29,30]. Working-related characteristics included year of employment, professional title, and setting.

### Statistical Analysis

CFA with diagonally weighted least squares estimation for ordinal data was conducted to verify the four-factor structure of PETS-C Brief previously reported [14], with full information maximum likelihood was used for handling missing data. The goodness of fit was determined by a combination of the following indicators: comparative fit index (CFI; ≥0.90), root mean square error of approximation (RMSEA; <0.08), and standardized root mean squared residual (SRMR; <0.08) [31]. We reported results of the *χ*^2^ test for descriptive purpose but not for evaluating the model fit (cutoff for a good fit: *P* >.05), because the result is always statistically significant in large samples [32]. Standardized factor loadings of 19 items on the four factors were evaluated, with a value ≥0.70 indicating a strong measure of the factor [27]. Cronbach α was calculated for internal consistency reliability. One-month test–retest reliability was determined by intraclass correlation coefficients (ICC) calculated based on two-way mixed-effects model with absolute agreement, by the following criteria: poor (<0.4), fair (0.4‐0.6), good (0.6‐0.75), and excellent (>0.75) [33]. Associations of sociodemographic and working-related characteristics with PETS-C Brief total scores and subscores of four factors were analyzed using multivariable linear regression, with complete case analysis was used for handling missing data. Known-group validity was assessed by examining associations of age and educational attainment with PETS-C Brief total scores. All analyses were conducted using STATA (version MP 15.1; StataCorp). *P*<.05 was considered statistically significant.

### Ethical Considerations

The study procedures were carried out in accordance with the Declaration of Helsinki. Participants were informed of the right to refuse to participate or to withdraw consent to participate at any time without reprisal by contacting the research team; all data were deidentified and no compensation was provided to participants. The research protocol was approved by the Shanghai Ninth Peoples Hospital, Shanghai Jiao Tong University School of Medicine Ethics Committee (SH9H2024-T99-3).

## Results

A total of 1482 nurses entered the study by scanning the QR code, of whom 49 were excluded due to incomplete survey, and 24 were excluded due to ineligibility. Table 1 shows participant characteristics. As a result, 1409 participants (n=1364, 96.8% female; mean age 34.6, SD 8.6 y) completed the survey, yielding a valid response rate of 95.1%. Around three-quarters (n=1044, 74.1%) held a undergraduate or higher educational degree.

**Table 1.** Participant characteristics (N=1409).

Characteristic	Participants (N=1409)
Female (ref: male), n (%)	1364 (96.8)
Age (years), mean (SD)	34.6 (8.6)
Educational attainment, n (%)	
College and below	365 (25.9)
Undergraduate	1019 (72.3)
Postgraduate	25 (1.8)
Marital status, n (%)	
Unmarried	462 (32.8)
Married	914 (64.9)
Divorced/widowed	33 (2.3)
Monthly household income (RMB, 1 USD≈7.0 RMB), n (%)	
<5,000	85 (6.0)
5,000-10,000	335 (23.8)
10,000-15,000	318 (22.6)
15,000-20,000	266 (18.9)
20,000-25,000	180 (12.8)
≥25,000	225 (16.0)
Years of employment, n (%)	
<5	330 (23.4)
5-10	285 (20.2)
≥10	794 (56.4)
Nurse (ref: Nurse manager and above), n (%)	987 (70.1)
Setting, n (%)	
Hospital, categorized by department	1215 (86.2)
Internal Medicine	307 (25.3)
Surgery	397 (32.7)
Gynecology and Obstetrics	169 (13.9)
Pediatrics	12 (1.0)
Emergency Medicine	26 (2.1)
Critical Care Medicine	97 (8.0)
Others	207 (17.0)
Community, n (%)	194 (13.8)

Table 2 shows the indicators of goodness of fit of the four-factor structure of the PETS-C Brief. The preliminary model fit was marginally acceptable (*χ*^2_11_^=1616.7, CFI=0.93, RMSEA=0.085, SRMR=0.064). The model was corrected based on the covariance modification indices to improve the model fit, with one pair of error terms with the largest indices (errors of item 13 and 14) covaried. The corrected model showed acceptable indicators of goodness of fit (*χ*^2^_8.8_=1277.9, CFI=0.95, RMSEA=0.074, SRMR=0.065).

**Table 2.** Indicators of goodness of fit of the four-factor structure of the Perception of eHealth Technology Scale in Chinese Brief (PETS-C Brief).

Indicator	With four-factor and 19 test items	Corrected model with errors of item 13 and 14 covaried
*χ*^*2[Table-fn T2_FN3]*^^a^ (*P*>.05)	1616.7 (*P*<.001)	1277.9 (*P*<.001)
*χ* ^*2*^ */df^b^*	11.1	8.8
CFI^c^ ≥0.90	0.93	0.95
RMSEA^d^ <0.08 (95% CI)	0.085 (0.081-0.088)	0.074 (0.071-0.078)
SRMR^e^ <0.08	0.064	0.065

a *χ*2, Chi-square.

b df, degrees of freedom.

c CFI, comparative fit index.

d RMSEA, root mean square error of approximation.

e SRMR, standardized root mean squared error.

Table 3 shows that standardized factor loadings ranged 0.69‐0.95 of each item on four factors of PETS-C. All items indicated a strong measure of the respective factors, apart from item 14 with factor loading of 0.69 marginally below the cutoff of 0.70.

**Table 3.** Standardized factor loadings of the Perception of eHealth Technology Scale in Chinese Brief (PETS-C Brief) with 4 factor and 19 items.

Item	Knowledge of eHealth technology	Perception of the advantages of eHealth technology	Perception of the advantages of eHealth technology	Perception of eHealth applications
Item 1	0.81	—^a^	—	—
Item 2	0.85	—	—	—
Item 3	0.95	—	—	—
Item 4	0.88	—	—	—
Item 5	—	0.88	—	—
Item 6	—	0.89	—	—
Item 7	—	0.76	—	—
Item 8	—	0.84	—	—
Item 9	—	—	0.86	—
Item 10	—	—	0.90	—
Item 11	—	—	0.85	—
Item 12	—	—	0.85	—
Item 13	—	—	0.71	—
Item 14	—	—	0.69	—
Item 15	—	—	—	0.78
Item 16	—	—	—	0.71
Item 17	—	—	—	0.85
Item 18	—	—	—	0.89
Item 19	—	—	—	0.83

a Not applicable.

Table 4 shows that the mean score of PETS-C Brief was 63.6 (SD 10.9). The four factors showed mean scores ranging 3.0‐3.7. PETS-C Brief and its four factors showed a good internal consistency (Cronbach α range=0.91‐0.93) and one-month test-retest reliability (ICC range 0.62‐0.73).

**Table 4.** Scores, internal consistency reliability, and 1-month test-retest reliability on the Perception of eHealth Technology Scale in Chinese Brief (PETS-C Brief) and four factors.

Variables	Score, mean (SD)	Cronbach α	Intraclass correlation coefficient (95% CI)
PETS-C Brief	63.6 (11.0)	0.91	0.70 (0.57-0.79)
Factor			
Knowledge of eHealth technology	3.4 (0.9)	0.92	0.67 (0.52-0.77)
Perception of the advantages of eHealth technology	3.7 (0.8)	0.91	0.62 (0.46-0.73)
Perception of the advantages of eHealth technology	3.0 (0.9)	0.93	0.73 (0.61-0.81)
Perception of eHealth applications	3.6 (0.7)	0.91	0.72 (0.59-0.80)

Table 5 shows the associations of sociodemographic and working-related characteristics with total scores and subscores of four factors, after mutual adjustment. Known-group validity was supported by inverse association of age with PETS-C Brief total scores (adjusted β=−0.20, 95% CI −0.32 to −0.07; *P*=.002) and positive association of educational attainment with PETS-C Brief total scores (*P* for trend=0.043). No significant associations were observed between working-related characteristics and PETS-C Brief total scores. Specifically, age was associated with subscores of knowledge (adjusted β=−0.02, 95% CI −0.03 to −0.01; *P*=.001) and perception of the disadvantages (adjusted β=−0.02, 95% CI −0.03 to −0.01; *P*=.001). Higher educational attainment was associated with subscores of perception of the advantages (*P* for trend=.007) and perception of eHealth applications (*P* for trend=.004). Higher monthly household income was also associated with subscores of perception of the advantages (*P* for trend=.01) and perception of eHealth applications (*P* for trend=.043). Years of employment was positively associated with subscores of knowledge (*P* for trend=.03). Compared with nurse manager and above, nurses reported lower subscores of perception of the advantages (adjusted β=−0.12, 95% CI −0.23 to −0.01; *P*=.03). Nurses working in the community setting than the hospital reported lower subscores of knowledge (adjusted β=−0.17, 95% CI −0.31 to −0.04; *P*=.01) and perception of the advantages (adjusted β=−0.13, 95% CI −0.25 to −0.01, *P*=.044).

**Table 5.** Sociodemographic and working-related characteristics associated with PETS-C Brief total scores and subscores of four factors.

Variables	Total		Knowledge of eHealth technology		Perception of the advantages of eHealth technology		Perception of the disadvantages of eHealth technology		Perception of eHealth applications	
	Adjusted β (95% CI)^a^	*P* value	Adjusted β (95% CI)	*P* value	Adjusted β (95% CI)	*P* value	Adjusted β (95% CI)	*P* value	Adjusted β (95% CI)	*P* value
Female (ref: male)	−2.89 (−6.16, 0.39)	.08	−0.19 (−0.45, 0.07)	.15	−0.14 (−0.36, 0.09)	.24	−0.16 (−0.42, 0.09)	.21	−0.12 (−0.34, −.10)	.29
Age	−0.20 (−0.32,‐0.07)	.002	−0.02 (−0.03,‐0.01)	.001	−0.01 (−0.02, 0.002)	.13	−0.02 (−0.03,‐0.01)	.001	−0.0004 (−0.009, 0.008)	.93
Educational attainment										
College and below	0	ref	0	ref	0	ref	0	ref	0	ref
Undergraduate	0.89 (−0.55, 2.32)	.23	0.013 (−0.10, 0.13)	.82	0.10 (0.004, 0.20)	.045	−0.03 (−0.14, 0.09)	.66	0.12 (0.02, 0.21	.02
Postgraduate	4.49 (0.01, 8.97)	.05	0.21 (−0.14, 0.57)	.24	0.23 (−0.08, 0.54)	.14	0.29 (−0.06, 0.64)	.11	0.19 (−0.11, 0.50)	.21
*P* for trend		.043		.46		.007		.84		.004
Marital status										
Unmarried	0	ref	0	ref	0	ref	0	ref	0	ref
Married	0.16 (−1.51, 1.84)	.85	0.05 (−0.08, 0.19)	.42	−0.03 (−0.15, 0.09)	.61	0.05 (−0.08, 0.18)	.45	−0.05 (−0.16, 0.07)	.41
Divorced/widowed	−0.44 (−4.53, 3.66)	.83	−0.03 (−0.35, 0.30)	.87	−0.11 (−0.39, 0.17)	.45	0.07 (−0.25, 0.39)	.68	−0.06 (−0.34, 0.22)	.67
Monthly household income (RMB, 1 USD=7.0 RMB)										
<5000	0	ref	0	ref	0	ref	0	ref	0	ref
5000‐10,000	0.92 (−1.71, 3.55)	.49	−0.01 (−0.21, 0.20)	.94	0.13 (−0.05, 0.31)	.15	−0.03 (−0.24, 0.17)	.77	0.12 (−0.05, 0.30)	.18
10,000‐15,000	−0.82 (−3.48, 1.85)	.55	−0.13 (−0.34, 0.08)	.23	0.17 (−0.02, 0.35)	.08	−0.24 (−0.45,‐0.03)	.02	0.10 (−0.08, 0.28)	.29
15,000‐20,000	0.89 (−1.87, 3.64)	.53	−0.03 (−0.25, 0.18)	.76	0.16 (−0.03, −.35)	.11	−0.09 (−0.31, 0.12)	.40	0.19 (0.005, 0.38)	.043
20,000‐25,000	−0.30 (−3.17, 2.58)	.84	−0.14 (−0.37, 0.08)	.22	0.14 (−0.06, −.34)	.16	−0.14 (−0.36, 0.09)	.24	0.10 (−0.09, 0.30)	.29
>25,000	1.53 (−1.30, 4.36)	.29	−0.02 (−0.24, 0.20)	.86	0.29 (0.10, 0.49)	.004	−0.11 (−0.34, 0.11)	.32	0.23 (0.03, 0.42)	.02
*P* for trend		.41		.63		.01		.35		.043
Years of employment										
<5	0	ref	0	ref	0	ref	0	ref	0	ref
5‐10	1.96 (−0.12, 4.04)	.06	0.14 (−0.02, 0.30)	.09	0.17 (0.03, 0.31)	.02	0.04 (−0.13, 0.20)	.66	0.10 (−0.04, 0.24)	.17
≥10	2.14 (−0.45, 4.73)	.11	0.25 (−0.04, 0.45)	.018	0.12 (−0.06, 0.30)	.19	0.10 (−0.10, 0.31)	.31	0.008 (−0.17, 0.18)	.93
*P* for trend		.18		.03		.24		.48		.99
Nurse (ref: nurse manger and above)	−1.53 (−3.14, 0.10)	.06	0.001 (−0.13, −.13)	.98	−0.12 (−0.23,‐0.01)	.03	−0.10 (−023, 0.03)	.12	−0.09 (−0.20, 0.02)	.12
Community setting (ref: hospital)	−1.25 (−2.98, 0.49)	.16	−0.17 (−0.31,‐0.04)	.01	−0.13 (−0.25,‐0.01)	.044	0.05 (−0.08, 0.19)	.43	−0.08 (−0.19, 0.04)	.21

a Mutually adjusted for sociodemographic and working-related characteristics.

## Discussion

### Principal Findings

Our validation study supported the four-factor structure of PETS-C Brief with satisfactory validity and reliability in Chinese nurses. The results of CFA showed a four-factor structure. Though the *χ*^2^ test was statistically significant, this has been common in large samples [32]. Other model fit indicators including comparative fit index (CFI), standardized root mean squared residual (SRMR), and root mean square error of approximation (RMSEA) were within the prespecified cutoff values, suggesting that the four-factor model was acceptable. The original scale had 40 items on six items for assessing perception of telemedicine in clinicians in Iran, including knowledge, perception of the advantages, perception of the disadvantages, perception of necessity, perception of ease of usage, and perception of security [20]. The scale was then adapted in English for assessing perception of telemedicine in health care practitioners in Saudi Arabia, and knowledge, advantages, disadvantages, necessity, issues affecting telemedicine, and effectiveness obtained from patient feedback were identified as six factors [34]. The English version had two different factors compared with the original one, which might be due to cultural differences in understanding telemedicine. The Spanish adapted version reduced the item number to 13 and identified four factors including knowledge, disadvantages, utility, and knowledge of security in Ecuadorian clinicians [35]. Compared with telemedicine focusing on remote health care services, eHealth technologies include a broader range including mobile health, electronic health record, wearable devices, and online health education resources. Nurse-led eHealth technology use has been increasingly promoted by the Chinese government [21,22]. In the Chinese context, Kwan and colleges modified the scale into PETS-C Brief for assessing perceptions of eHealth technologies in nurses, and knowledge, perception of the advantages, perception of the disadvantages, and perception of eHealth applications were identified as four factors of the scale [14]. Our study complemented the study by the use of a large sample and confirmation of the four-factor structure. Nevertheless, more studies in nurse populations are warranted as the scale was less examined in this group than in clinicians.

Internal consistency reliability of PETS-C brief and its four factors were good in our study. Specifically, Cronbach α of the scale was higher than those conducted in Chinese nurses previously [11,14]. Changes in the sociocultural environment over years and the increased recognition of eHealth may have affected the way nurses understood and responded to the scale items, which in turn may have affected the internal consistency of the scale. Our study complemented previous findings by supporting one-month test–retest reliability of PETS-C Brief and its four factors, showing that the scale could be reliable over a short time. However, test–retest reliability findings must be interpreted with caution because of the relatively small sample size (n=123) and short time interval. Longitudinal studies following more nurses are needed to confirm the test–retest reliability of PETS-C Brief.

Known-group validity was supported by higher PETS-C Brief total scores associated with younger age and higher educational attainment. These findings were consistent with studies on digital divide in nurses showing that age [25,26,36] and educational attainment [11,24] could influence nurses’ perception of eHealth technologies. The study using PETS-C Brief in Chinese nurses further confirmed that higher educational attainment was associated with higher PETS-C Brief total scores [11]. Specifically, age was inversely associated with subscores of knowledge and perception of the disadvantage of eHealth technologies. Younger nurses may have earlier exposure in daily life and work, hence becoming more tech-savvy [36]. This may increase their knowledge and critical opinions of eHealth technologies. Educational attainment was positively associated with subscores of perception of the advantages and perception of eHealth applications. These findings were consistent with previous results on higher levels of digital literacy and comfort with technology adoption reported by nurses with higher educational attainment [37]. Nurses with higher educational attainment may have more trainings in use of eHealth technologies for clinical, research, and administration purposes, hence increasing their perceptions of advantages and eHealth applications. These findings supported the TAM, indicating that individual experience and training could influence perception of technologies [17]. Intervention and training programs can be delivered to nurses with older age and lower educational attainment for promoting eHealth technology use.

This study had several limitations. First, the original version of PETS-C Brief was initially developed to assess perceptions of telemedicine in Iranian clinicians, containing 40 items on six factors [20]. The scale was then adapted to 19 items on four factors in Chinese nurses through translation, item reduction, and validation [14]. Though poorly performing items were reduced and the new scale structure has been validated, differences in professional background, culture, and technology exposure may affect the meaning of items and factors. Second, the PETS-C Brief measures a broader concept of eHealth, which might reduce its usefulness for guiding targeted interventions adopting specific technologies, such as telemedicine. Third, convergent validation was unclear. Future studies may analyze correlations of PETS-C Brief with other widely used instruments such as TAM for confirming the convergent validation. such as instruments based on TAM. Fourth, participants were recruited using convenience sampling, and peer sharing or referral was used to increase the response rate. This may lead to mutual introduction, referring to the inclusion of a group of people with similar views, resulting in selective bias. Duplicate submissions by the same participant using different devices cannot be avoided. However, telephone numbers were checked, and no duplicate number was found. Future studies with random sampling are needed. Fifth, educational attainment and monthly household income were collected as categorical data, which inform future studies using continuous variables for providing more details. Last, all data were self-reported, which could be subjected to recall bias.

### Conclusions

Our validation study supported the four-factor structure of PETS-C Brief with satisfactory validity and reliability in Chinese nurses. With the pervasion of advanced eHealth technologies, PETS-C Brief can be a useful tool for continuously assessing Chinese nurses’ perception of eHealth technologies.

## Abbreviations

CFA: confirmatory factor analysis
CFI: comparative fit index
EFA: exploratory factor analysis
eHEALS: eHealth Literacy Scale
ICC: intraclass correlation coefficients
PETS-C Brief: Perception of eHealth Technology Scale in Chinese Brief
RMSEA: root mean square error of approximation
SRMR: standardized root mean square residual
TAM: technology acceptance model
